# Possible Resolution of Food Allergies Following Tocilizumab-Induced Remission of Juvenile Idiopathic Arthritis

**DOI:** 10.7759/cureus.79442

**Published:** 2025-02-22

**Authors:** Israel Magen, Rey Biton, Majd Khateeb, Eli Magen

**Affiliations:** 1 Medicine, Assuta Ashdod University Medical Center, Ashdod, ISR; 2 Internal Medicine, Assuta Ashdod University Medical Center, Ashdod, ISR

**Keywords:** food allergy, ige-mediated hypersensitivity, il-6 inhibition, jia, juvenile idiopathic arthritis, tocilizumab

## Abstract

This case report describes a unique instance of a four-year-old Ashkenazi Jewish male with systemic juvenile idiopathic arthritis (JIA) and severe IgE-mediated food allergies who achieved complete remission of both conditions following treatment with tocilizumab, an IL-6 receptor antagonist. The patient initially presented with anaphylactic reactions to peanuts, eggs, and cow’s milk, confirmed by positive skin prick tests and elevated specific IgE (sIgE) levels. Systemic JIA symptoms, including daily fever spikes, evanescent rash, and polyarticular arthritis, developed shortly thereafter. After inadequate response to standard therapies, tocilizumab was initiated, resulting in complete remission of JIA symptoms and unexpected resolution of food allergies. Repeat allergological evaluations, including skin prick tests, sIgE levels, and controlled oral food challenges, confirmed sustained tolerance to previously allergenic foods. The dual remission observed in this case suggests that interleukin-6 (IL-6) inhibition may modulate shared immunological pathways underlying autoimmune and allergic diseases. IL-6 is a key cytokine in both conditions, promoting Th17-mediated inflammation in JIA and Th2-mediated allergic responses, including IgE production and mast cell activation. While the spontaneous resolution of food allergies, particularly to milk and eggs, is well-documented in pediatric populations, the resolution of peanut allergy, in this case, is highly unusual and temporally associated with tocilizumab treatment. This case raises the possibility that IL-6 inhibition may play a role in modulating autoimmune and allergic responses, warranting further investigation into the interplay between these pathways.

## Introduction

Systemic juvenile idiopathic arthritis (JIA) is a distinct category of juvenile idiopathic arthritis characterized by systemic inflammation and is now recognized as an autoinflammatory disease rather than a classic autoimmune disorder [1]. The coexistence of autoimmune and allergic diseases in pediatric patients is increasingly recognized. For example, children with JIA have a 1.5- to two-fold higher prevalence of IgE-mediated food allergies compared to the general pediatric population [2].

Systemic JIA is driven primarily by dysregulation of the innate immune system, with elevated levels of pro-inflammatory cytokines such as interleukin-1 (IL-1), interleukin-6 (IL-6), and interleukin-18 (IL-18), and minimal involvement of adaptive immune mechanisms such as autoreactive T-cells or autoantibodies [3]. Current consensus guidelines recommend IL-1 and IL-6 inhibitors (e.g., anakinra and tocilizumab) as first- or second-line therapies for JIA, given their efficacy in controlling systemic inflammation and preventing disease complications [4]. In addition to its role in autoinflammatory diseases, IL-6 has been implicated in the pathogenesis of allergic diseases. IL-6 promotes Th2 differentiation, enhances IgE production, and contributes to mast cell activation, all of which are central to developing IgE-mediated food allergies [5]. This dual role of IL-6 in both autoinflammatory and allergic pathways provides a compelling rationale for investigating the potential impact of IL-6 inhibition on food allergy outcomes, as illustrated in this case report.

## Case presentation

A four-year-old Ashkenazi Jewish male presented in February 2023 with a severe anaphylactic reaction after ingesting peanut-containing snacks (Bamba) and undercooked eggs. The patient's food allergies first manifested at 18 months of age, with the initial anaphylactic reaction occurring after cow's milk ingestion. Subsequent episodes were attributed to individual ingestion of eggs and peanuts, respectively. The presenting episode in February 2023 was unique in involving simultaneous exposure to peanuts and undercooked eggs. The systemic allergic reactions to these foods were consistent with grade 3 anaphylaxis according to the World Allergy Organization (WAO) Systemic Allergic Reaction Grading System [6]. Symptoms included rapid onset of generalized urticaria, angioedema of the lips and eyelids, persistent cough, wheezing, and hypotension. Each episode required immediate intramuscular epinephrine administration and emergency room treatment. Given this history of severe, life-threatening reactions and the clear temporal relationship between allergen exposure and symptom onset, the risk of conducting food challenges was deemed to outweigh the potential benefits at that time.

The patient had no history of other atopic disorders, such as atopic dermatitis or airway allergies. Family history was negative for atopic diseases, including food allergies, asthma, and eczema. There was no history of consanguinity in the patient's family. Allergy evaluation confirmed IgE-mediated hypersensitivity to these foods, evidenced by positive skin prick tests and elevated specific IgE (sIgE) levels measured via ImmunoCAP (Table 1). 

**Table 1:** Laboratory and allergy evaluation sIgE: specific immunoglobulin E; ImmunoCAP: Immuno Solid-phase Allergen Chip; CRP: C-reactive protein; ANA: antinuclear antibodies; RF: rheumatoid factor; CMV IgM: cytomegalovirus immunoglobulin M; EBV VCA IgM: Epstein-Barr virus viral capsid antigen immunoglobulin M

Parameter	Initial value	After six months	Reference range
Skin prick test (cow's milk)	6 mm wheal/14 mm flare	Negative (0 mm wheal)	Negative (0 mm wheal)
Skin prick test (egg white)	5 mm wheal/12 mm flare	Negative (0 mm wheal)	Negative (0 mm wheal)
Skin prick test (peanut)	8 mm wheal/15 mm flare	Negative (0 mm wheal)	Negative (0 mm wheal)
sIgE cow's milk (ImmunoCAP)	5.2 kU/L	<0.35 kU/L	<0.35 kU/L
sIgE egg white (ImmunoCAP)	2.8 kU/L	<0.35 kU/L	<0.35 kU/L
sIgE peanut (ImmunoCAP)	14.5 kU/L	<0.35 kU/L	<0.35 kU/L
Total IgE	358 kU/L		2-57 kU/L
White blood cell count	16,570/μL	8,420/μL	5,000-14,500/μL
Platelet count	472,000/μL	281,000/μL	150,000-400,000/μL
Hemoglobin	10.1 g/dL	12.7 g/dL	11.5-14.5 g/dL
Ferritin	679 ng/mL	53 ng/mL	7-140 ng/mL
Erythrocyte sedimentation rate (ESR)	62 mm/hr	11 mm/hr	0-20 mm/hr
C-reactive protein (CRP)	24.8 mg/dL	0.9 mg/dL	<0.5 mg/dL
Antinuclear antibodies (ANA)	Negative	-	Negative
Rheumatoid factor (RF)	Negative	-	Negative
CMV IgM	Negative	-	Negative
EBV VCA IgM	Negative	-	Negative

The patient's systemic JIA symptoms began in early February 2022, shortly after the anaphylactic episode. The fever pattern was characteristic of systemic JIA, with daily spikes above 39°C that persisted for over two weeks. Joint inflammation, primarily affecting the knees, ankles, and wrists, developed within three days of fever onset. A transient salmon-pink rash appeared on the trunk and limbs during fever spikes. The diagnosis of systemic juvenile idiopathic arthritis (sJIA) was established based on the International League of Associations for Rheumatology (ILAR) classification criteria, which include daily fever spikes ≥39°C for at least two weeks, arthritis, and characteristic systemic features such as an evanescent rash [7]. Comprehensive laboratory and imaging studies ruled out infectious and other inflammatory conditions. Infectious and other inflammatory conditions were ruled out through laboratory and imaging studies. Initial management with non-steroidal anti-inflammatory drugs (NSAIDs) (100 mg/5 mL ibuprofen syrup, administered 5 mL every six hours as needed for fever and joint pain) and corticosteroids (prednisone syrup 5 mg/5 mL, administered 18 mg/day qd and tapered over eight weeks) yielded a suboptimal clinical response, necessitating the initiation of tocilizumab (120 mg IV) in May 2022. Monthly infusions were administered, gradually improving JIA's systemic and articular manifestations. By the sixth infusion, complete remission of JIA symptoms was achieved, prompting continued treatment for a total of 12 infusions until May 2023. The patient met the American College of Rheumatology (ACR) provisional criteria for clinical remission in JIA, including the absence of systemic symptoms, active arthritis, and normalization of inflammatory markers [8].

The decision to continue tocilizumab for 12 months was based on the ACR guidelines for treating systemic JIA, which recommend maintaining treatment for at least six to 12 months after achieving clinical remission to minimize the risk of relapse [9].

The patient's food allergies also showed unexpected resolution during tocilizumab treatment. Six months after initiating tocilizumab therapy, the parents reported that the patient had inadvertently consumed small amounts of cow’s milk, egg, and peanut without any adverse reactions. Encouraged by this observation, the parents continued introducing these foods in small quantities at home, and the patient remained symptom-free. Repeat allergological evaluations were performed to assess the patient’s allergic status objectively. Skin prick tests (SPTs) for cow’s milk, egg white, and peanut, which were previously positive (6 mm, 5 mm, and 8 mm wheal, respectively), now showed complete resolution (0 mm wheal/0 mm flare). Similarly, serum-specific IgE (sIgE) levels, which were initially elevated (cow’s milk: 5.2 kU/L; egg white: 2.8 kU/L; peanut: 14.5 kU/L), had normalized to <0.35 kU/L for all three allergens. Given these findings, controlled oral food challenges (OFCs) were conducted under medical supervision to confirm tolerance. The patient successfully tolerated age-appropriate servings of cow’s milk (250 mL), scrambled egg (50 g), and peanut butter (10 g) without any clinical reactions. These results, supported by the resolution of SPTs, normalization of sIgE levels, and successful OFCs, confirm the sustained resolution of the patient’s food allergies. The timeline of symptoms and treatment is presented in Table 2.

**Table 2:** Timeline of symptoms and treatment NSAIDs: non-steroidal anti-inflammatory drug; JIA: juvenile idiopathic arthritis; ILAR: International League of Associations for Rheumatology

Date	Event	Symptoms/Findings	Management/Treatment
July 2019	First anaphylactic reaction (cow’s milk)	Generalized urticaria, angioedema, wheezing, vomiting	Intramuscular epinephrine, emergency room treatment
September 2020	Second anaphylactic reaction (eggs)	Generalized urticaria, angioedema, wheezing, hypotension	Intramuscular epinephrine, emergency room treatment
July 2021	Third anaphylactic reaction (peanuts)	Generalized urticaria, angioedema, wheezing, vomiting	Intramuscular epinephrine, emergency room treatment
February 2022	Severe anaphylactic reaction (peanuts and undercooked eggs)	Generalized urticaria, angioedema, persistent cough, wheezing, vomiting, hypotension (Grade 3 anaphylaxis)	Intramuscular epinephrine, emergency room treatment
February 2022	Systemic JIA symptoms onset ILAR classification criteria met	Daily fever spikes ≥39°C, evanescent salmon-pink rash, polyarticular arthritis (knees, ankles, wrists)	NSAIDs (ibuprofen), corticosteroids (prednisone)
May 2022	Initiation of tocilizumab	Refractory JIA symptoms	Tocilizumab (120 mg IV monthly infusions)
November 2022	Unexpected resolution of food allergies	No adverse reactions to inadvertent consumption of cow’s milk, egg, and peanut	Continued introduction of allergenic foods at home
January 2023	Controlled oral food challenges (OFCs)	Successful tolerance of cow’s milk (250 mL), scrambled egg (50 g), peanut butter (10 g)	Confirmed sustained resolution of food allergies
May 2023	Completion of tocilizumab therapy	Complete remission of JIA symptoms	A total of 12 tocilizumab infusions administered

## Discussion

This case report presents a novel observation of concurrent remission of systemic JIA and IgE-mediated food allergies following IL-6 blockade with tocilizumab. While tocilizumab's primary mechanism involves inhibiting IL-6 signaling, leading to reduced inflammation in autoimmune diseases [10], its potential role in modulating allergic responses warrants further investigation.

The diagnosis of peanut, hen's egg, and cow's milk allergies was initially based on the patient's history of anaphylactic reactions, positive skin prick tests (SPTs), and elevated specific IgE (sIgE) levels measured via ImmunoCAP. Before tocilizumab treatment, oral food challenges were not performed due to the severity of previous reactions and ethical considerations. The patient's total serum IgE level at initial presentation was 358 kU/L (reference range: < 57 kU/L). While it is acknowledged that egg and milk allergies often resolve spontaneously in young children, the patient's peanut allergy was considered persistent based on the severity of reactions and consistently high sIgE levels. The patient's peanut allergy was initially considered persistent based on the severity of prior reactions and consistently high sIgE peanut levels, which were measured at diagnosis (February 2022: 14.5 kU/L) and at follow-up time points (August 2022: 12.8 kU/L).

To confirm the resolution of food allergies post-tocilizumab treatment, we conducted controlled oral food challenges under medical supervision. These challenges were performed sequentially for milk, egg, and peanut, all of which were tolerated without adverse reactions.

The patient's peanut allergy was evidenced by a severe anaphylactic reaction following ingestion of peanut-containing snacks (Bamba) in February 2022, which co-occurred with egg ingestion. Previously isolated exposure to peanuts also resulted in allergic reactions, supporting the diagnosis of peanut allergy independent of other food allergens.

We conducted a comprehensive immunological workup to rule out potential underlying human inborn immunity errors that could explain JIA's coexistence and severe food allergies. This included the evaluation of lymphocyte subsets, immunoglobulin levels (IgG, IgA, IgM), and complement levels (C3, C4), which were normal. The patient did not exhibit clinical features typically associated with dedicator of cytokinesis 8 (DOCK8) deficiency. This comprehensive evaluation suggests that the patient's presentation of concurrent JIA and severe food allergies was not attributable to a known underlying human inborn error of immunity. 

The patient's Ashkenazi Jewish heritage is a population with well-documented genetic predispositions to autoimmune conditions, whereas their link to allergic conditions is much less established [11]. While the relationship between Ashkenazi Jewish ancestry and allergic diseases is not fully understood, some studies suggest that genetic and environmental factors may contribute to a higher prevalence of atopic conditions in this population. Genetic variants in immune-related genes, such as FCER1A, have been associated with allergic sensitization [12].

This case report presents a novel observation of concurrent remission of systemic JIA and IgE-mediated food allergies following IL-6 blockade with Tocilizumab. The unexpected resolution of food allergies in this patient following treatment with tocilizumab highlights the potential dual role of IL-6 inhibition in modulating both autoimmune and allergic pathways. IL-6 is a key driver of Th17 differentiation, critical in the pathogenesis of autoimmune diseases like systemic JIA. Th17 cells produce pro-inflammatory cytokines such as IL-17 and IL-22, contributing to systemic inflammation and tissue damage [10, 13]. Interestingly, IL-6 also promotes Th2 differentiation, central to allergic responses. Th2 cells produce IL-4, IL-5, and IL-13 cytokines, which drive IgE production, eosinophil activation, and mast cell proliferation [14]. IL-6 is essential for B cell maturation and immunoglobulin class switching, including the production of IgE [15]. IL-6 potentiates mast cell activation and the release of inflammatory mediators such as histamine, leukotrienes, and prostaglandins, which are key players in anaphylactic reactions [16]. Moreover, IL-6 blockade can enhance the suppressive function of Tregs, thereby restoring immune tolerance to allergens [17]. By modulating these immune pathways, tocilizumab might have attenuated the allergic responses in this patient.

Existing literature provides limited evidence of its efficacy in treating allergic conditions with tocilizumab. A notable study investigated the impact of tocilizumab on allergen-induced bronchoconstriction in patients with mild allergic asthma. A single dose of tocilizumab did not significantly prevent allergen-induced bronchoconstriction, suggesting the limited effectiveness of IL-6 blockade in this context [18]. In contrast, our case presents a unique scenario with dual remission and concurrent resolution of autoimmune and allergic diseases with IL-6 inhibition.

We acknowledge that most children outgrow milk and egg allergies by age three to five and that peanut allergies, while less common, can also be outgrown in some cases [19]. Moreover, a causal relationship cannot be established in a single case report, but we would like to highlight several unique aspects of this case that make it noteworthy. A temporal association was noted; the resolution of food allergies coincided with the administration of tocilizumab, which is an intriguing observation worthy of reporting. The patient's food allergy going away was confirmed by objective information like repeat allergological tests that showed negative skin prick tests, normalization of serum-specific IgE levels, and successful oral food challenges. The clinical course of this case was unusual since the patient had severe, persistent food allergies that resolved unexpectedly during tocilizumab treatment, which is not typical of the natural history of food allergy outgrowth.

## Conclusions

While the resolution of food allergies, in this case, may be coincidental or related to the natural history of food allergy outgrowth, the temporal association with tocilizumab treatment raises the possibility of a modulatory effect on allergic pathways. This case should, therefore, be viewed as hypothesis-generating, suggesting a potential role for IL-6 inhibition in modulating allergic pathways. The patient’s Ashkenazi Jewish heritage may also be relevant, as genetic predispositions in this population could influence responses to IL-6 inhibition. Further studies, including larger cohorts and mechanistic investigations, are needed to explore this possibility and to determine whether tocilizumab or other IL-6 inhibitors could have therapeutic implications for patients with coexisting autoimmune and allergic conditions.
